# SURVIVAL AFTER DEMENTIA DIAGNOSIS: VARIATION BY RACE/ETHNICITY AND GEOGRAPHIC FACTORS

**DOI:** 10.1093/geroni/igac059.1397

**Published:** 2022-12-20

**Authors:** Haiqun Lin, Olga Jarrín, Bei Wu, Paul Duberstein, Anum Zafar, Maria Lopez

**Affiliations:** Rutgers University, Newark, New Jersey, United States; Rutgers, The State University of New Jersey, New Brunswick, New Jersey, United States; New York University, New York, New York, United States; Rutgers School of Public Health, Piscataway, New Jersey, United States; Rutgers, The State University of New Jersey, New Brunswick, New Jersey, United States; Rutgers, The State University of New Jersey, New Brunswick, New Jersey, United States

## Abstract

This study describes racial and ethnic variation in dementia diagnosis and survival time between diagnosis and death for Medicare beneficiaries aged 50 years and older who died in 2018 (n=1,998,282). The prevalence of diagnosed dementia was higher among non-Hispanic white (45.7%), Black (45.5%), and American Indian/Alaska Native (44.1%) decedents compared to Asian American/Pacific Islander (42.7%), and Hispanic (38.5%) decedents. Median survival time in years was shorter for American Indian/Alaska Native (2.51, IQR 0.7-6.0) and white (2.85, IQR 0.8-6.3) beneficiaries, and longer for Asian American/Pacific Islander (3.36, IQR 1.0-7.3), Black (3.38, IQR 0.9-7.4), and Hispanic (3.83, IQR 1-8.2) beneficiaries. Median survival time also varied by geography and was shortest for Hispanic decedents in Arizona (2.37, IQR 0.6-6.0) and New Mexico (2.92, IQR 0.8-7.0) compared to New Jersey (4.85, IQR 1.5-8.9) and Puerto Rico (5.45, IQR 1.1-12.8). Among Black decedents, survival was longest in New Jersey (3.80, 1.1-7.9) and Arkansas (4.22, 1.0-8.5).

